# Supplementary material for: Self-evolving photonic crystals for ultrafast photonics
Source: Nat Commun. 2023 Jan 27;14:50. doi: 10.1038/s41467-022-35599-2 (PMC9883472; doi:10.1038/s41467-022-35599-2)
Supplement: Supplementary file 1 — Supplementary Information [file 41467_2022_35599_MOESM1_ESM.pdf]

SUPPLEMENTARY INFORMATION

Self-evolving photonic crystals for ultrafast photonics

Takuya Inoue,[1,*,†] Ryohei Morita,[2,*] Kazuki Nigo,[1] Masahiro Yoshida,[2] Menaka De Zoysa,[1] Kenji Ishizaki,[1] and Susumu Noda[1,†]

[1]Photonics and Electronics Science and Engineering Center, Kyoto University,

[2]Department of Electronic Science and Engineering, Kyoto University,

Kyoto 615-8510, Japan

*These authors contributed equally to this work.

†To whom correspondence should be addressed.

E-mail: t_inoue@qoe.kuee.kyoto-u.ac.jp, snoda@kuee.kyoto-u.ac.jp.

Supplementary Note 1. Device structure and its photonic band diagram

Photonic-crystal surface-emitting lasers (PCSELs) are lasers that utilize a two-dimensional standing-wave resonance at a singularity point (Γ point, etc.) of the photonic band for lasing oscillation. Figure S1a shows a cross-section view of the device considered in this work, where a GaAs double-lattice photonic crystal (PC) layer is located near the active layer (InGaAs/AlGaAs quantum wells) and is sandwiched by p- and n-AlGaAs cladding layers. The thickness and refractive index of each layer is provided in Table S1. The light emitted downward from the photonic crystal layer is reflected at the p-type distributed Bragg reflector (DBR) below the p-cladding layer and then interferes with the upward emission. Figure S1b shows an enlarged top view of a double-lattice structure composed of an elliptic and circular hole, which possesses reflection symmetry along the line of y=x. As detailed in our recent theoretical work [S1], by tuning the lattice separation of d and hole-size balance $2x$ in Fig. S1b, we can arbitrarily control the magnitude of the in-plane optical feedback inside the photonic crystal through destructive interference between 180° and 90° wave diffractions (i.e. Hermitian coupling coefficient $\kappa_{1D} + \kappa_{2D-}$). In addition, we can also control the magnitude of the optical couplings with accompanying vertical radiation loss (i.e. non-Hermitian coupling coefficient $i\mu$) by adjusting the thickness of the p-cladding layer, which influences the phase difference between the light emitted upward and the light reflected from the DBR.

As a result, we can stably select one mode among the four band-edge modes as the lasing mode at the Γ point of the photonic crystal, as well as increase the radiation constant difference ($\Delta\alpha_v$) between the fundamental mode and the other higher-order modes even for ultra-large-area (>3 mm) devices [S1]. In this work, we design a double-lattice photonic crystal with moderate Hermitian and non-Hermitian coupling coefficients (d=0.260a, 2x=3.1 nm, which corresponds to $\kappa_{1D}+\kappa_{2D-}=-71-16i$ cm^{-1} and $\mu=85$ cm^{-1}) to obtain single-mode lasing in a 1-mm-diameter device. Figure S1c shows the calculated photonic band diagram for the designed double-lattice photonic crystal, alongside the radiation constants of the four band-edge modes in an infinite structure. The mode with the lowest frequency (mode A) is the lasing mode owing to its smallest radiation constant, and the mode-gap for mode A exists above the band-edge frequency.

Table S1| Structural parameters of the designed device.

Layer	Thickness (nm)	Refractive index
n-cladding (AlGaAs)	1100	3.38
AlGaAs	300	3.45
Active (InGaAs/AlGaAs)	(10/20)×3	3.58/3.45
AlGaAs	25	3.27
GaAs	90	3.55
PC	160	n_{pc}
p-cladding (AlGaAs)	970	3.32
DBR (AlGaAs/AlGaAs)	(68/78) ×14	3.47/3.01
Contact (GaAs)	300	3.55

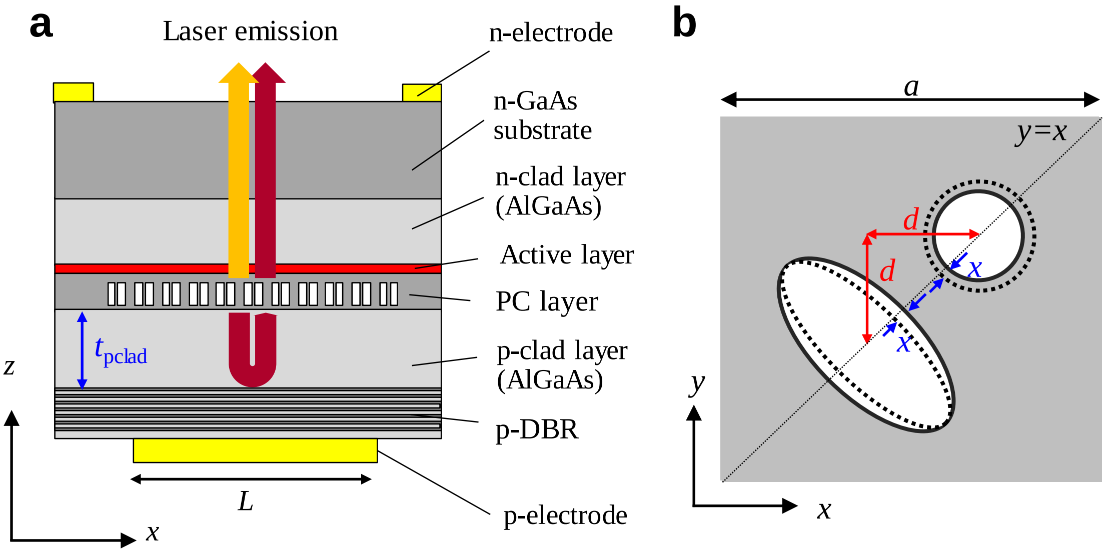

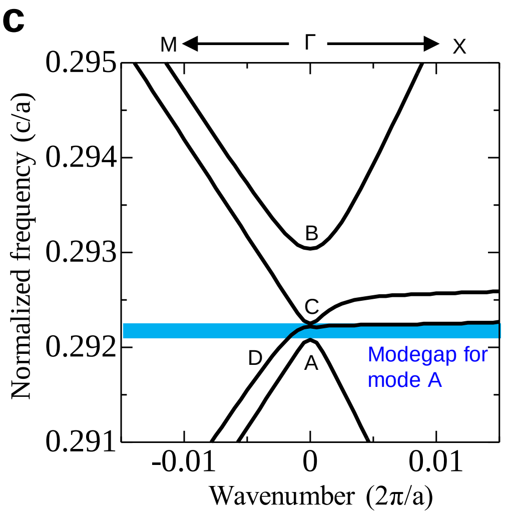

Mode	α_v (cm^{-1})
B	154.7
C	330.8
D	171.5
**A (lasing)**	**8.3**

Figure S1| Device structure and photonic band diagram. a, Cross section of a typical PCSEL. **b,** Schematic of a double-lattice photonic crystal with elliptic and circular air holes. **c,** Calculated photonic band diagram of the designed double-lattice photonic crystal. The radiation constants of the four band-edge modes are shown in the table.

Supplementary Note 2. Method of transient analysis of self-evolving photonic crystals

A transient analysis of the self-evolving photonic crystals was performed using time-dependent three-dimensional coupled-wave theory (3D-CWT) [S2]. The rate equations for the complex amplitudes of the basic waves inside the photonic crystal and the carrier density are expressed as follows;

$$\frac{\partial}{\partial t}\begin{pmatrix} R_x \\ S_x \\ R_y \\ S_y \end{pmatrix} = \frac{c}{n_\mathrm{g}}\left[-i\frac{2\pi}{\lambda}\Gamma\Delta n(N) + \frac{\Gamma g(N)-\alpha_\mathrm{in}}{2}\right]\begin{pmatrix} R_x \\ S_x \\ R_y \\ S_y \end{pmatrix} + \frac{ic}{n_\mathrm{g}}\mathbf{C}\begin{pmatrix} R_x \\ S_x \\ R_y \\ S_y \end{pmatrix} - \frac{c}{n_\mathrm{g}}\begin{pmatrix} \partial R_x/\partial x \\ -\partial S_x/\partial x \\ \partial R_y/\partial y \\ -\partial S_y/\partial y \end{pmatrix} + \begin{pmatrix} f_1 \\ f_2 \\ f_3 \\ f_4 \end{pmatrix}, \quad \text{(S1)}$$

$$\frac{\partial N}{\partial t} = \frac{J}{ed_\mathrm{QW}} - \frac{N}{\tau_\mathrm{c}} - \frac{c}{n_\mathrm{g}}g(N)U + D\nabla^2 N\,. \quad \text{(S2)}$$

Here, R_x, S_x, R_y, S_y represent the position- and time-dependent amplitudes of the four basic waves propagating in the $+x$, $-x$, $+y$, and $-y$ directions, respectively, and N is the carrier density. **C** is a 4×4 matrix which represents the Hermitian and non-Hermitian cross-couplings among the basic waves [S1,S3], which is determined by the Fourier coefficients of the photonic-crystal layer. n_g is the group refractive index of the multi-layered structure, Γ is the optical confinement factor inside the active layer, $\Delta n(N)$ and $g(N)$ are the carrier-dependent refractive-index change and optical gain, respectively, α_in is the internal material loss, f_i (i=1–4) are the spontaneous emission terms, J is the current density, d_QW is the thickness of the active layer, τ_c is the carrier lifetime, and D is the carrier diffusion

coefficient. U is the photon density inside the active layer, which is given by the following equation:

$$U = \Gamma \frac{2\varepsilon_0 n_{\mathrm{eff}} n_{\mathrm{g}}}{\hbar \omega d_{\mathrm{QW}}} \left[|R_x|^2 + |S_x|^2 + |R_y|^2 + |S_y|^2 \right]. \tag{S3}$$

In Eqs. (S1)-(S3), both the electric field amplitudes and the carrier density (and the other related parameters) depend on the position **r**. The first, second, and third terms on the right side of Eq. (S1) denote carrier-induced frequency and gain change, mutual couplings among the four basic waves, and spatial propagation, respectively. To consider a graded photonic crystal with lattice-point shifts (Δx, Δy), we employed the following position-dependent coupling-wave matrix $\mathbf{C}'$ in each microscopic region:

$$\mathbf{C}' = \begin{pmatrix} c_{11} & c_{12}\exp(i2\beta_0\Delta x) & c_{13}\exp(i\beta_0(\Delta x - \Delta y)) & c_{14}\exp(i\beta_0(\Delta x + \Delta y)) \\ c_{21}\exp(-i2\beta_0\Delta x) & c_{22} & c_{23}\exp(-i\beta_0(\Delta x + \Delta y)) & c_{24}\exp(-i\beta_0(\Delta x - \Delta y)) \\ c_{31}\exp(-i\beta_0(\Delta x - \Delta y)) & c_{32}\exp(i\beta_0(\Delta x + \Delta y)) & c_{33} & c_{34}\exp(i2\beta_0\Delta y) \\ c_{41}\exp(-i\beta_0(\Delta x + \Delta y)) & c_{42}\exp(i\beta_0(\Delta x - \Delta y)) & c_{43}\exp(-i2\beta_0\Delta y) & c_{44} \end{pmatrix}, \tag{S4}$$

where β_0 represents the wavenumber of the basic waves and

$$\mathbf{C} = \begin{pmatrix} c_{11} & c_{12} & c_{13} & c_{14} \\ c_{21} & c_{22} & c_{23} & c_{24} \\ c_{31} & c_{32} & c_{33} & c_{34} \\ c_{41} & c_{42} & c_{43} & c_{44} \end{pmatrix} \tag{S5}$$

represents the original coupled-wave matrix without lattice-constant gradation.

In our simulation, we assumed a uniform current distribution inside the current-injection section for simplicity. The spontaneous emission terms f_i are given by random Gaussian noise, whose correlations are given by

$$\left\langle f_i^*(\mathbf{r},t)\, f_j(\mathbf{r}',t')\right\rangle = C'\frac{\beta N}{\tau_{\mathrm{c}}}\delta_{ij}\delta(\mathbf{r}-\mathbf{r}')\delta(t-t')$$
$$C' = \left(\frac{2\varepsilon_0 n_{\mathrm{eff}} n_{\mathrm{g}}}{\hbar\omega d_{\mathrm{QW}}}\right)^{-1} \quad , \qquad \text{(S6)}$$

where β is the spontaneous emission factor.

The carrier-dependent optical gain $g(N)$ and refractive-index change $\Delta n(N)$ of the active layer (InGaAs/AlGaAs quantum wells) at the lasing wavelength were calculated using the method explained in Ref. S4, where the occupation probabilities in the conduction and valence bands of the active layer were calculated in advance using *k-p* perturbation theory [S5]. The calculated gain and index change were fitted using the following linear fractional functions of the carrier density:

$$g(N) = \frac{g_{\max}\left(N - N_{\mathrm{tr}}\right)}{N + \left[g_{\max}/\left(-g_0\right)\right]N_{\mathrm{tr}}}\cdot\frac{1}{1+\varepsilon U}, \qquad \text{(S7)}$$

$$\Delta n(N) = \frac{\Delta n_{\max} N}{N + N_{\mathrm{tr2}}}. \qquad \text{(S8)}$$

Here, N_{tr} is the transparency carrier density, $g_{\max}$ is the maximum gain, $(-g_0)$ is the absorption coefficient when there are no carriers, and ε is the gain suppression factor considering spectral hole burning and carrier heating [S6, S7]. $\Delta n_{\max}$ and N_{tr2} in Eq. (S8) are fitting parameters for the calculated refractive-index change.

The electric field of the radiative waves emitted vertically from each section of the device ($E_{x,\mathrm{rad}}$, $E_{y,\mathrm{rad}}$) can be calculated from the amplitude of the four basic waves [R_x,

S_x, R_y, S_y] as follows;

$$\begin{aligned} E_{x,\text{rad}} &= -\left(\sqrt{2\mu}R_y + \sqrt{2\mu}e^{i\theta_{\text{pc}}}S_y\right) \\ E_{y,\text{rad}} &= \sqrt{2\mu}R_x + \sqrt{2\mu}e^{i\theta_{\text{pc}}}S_x \end{aligned} . \quad \text{(S9)}$$

In Eq. S9, μ represents the magnitude of the non-Hermitian coupling coefficient of the photonic crystal, which corresponds to the imaginary part of the diagonal terms of $\mathbf{C}$, and θ_{pc} represents the phase of the non-Hermitian 180°-coupling [S1]. In our designed double-lattice photonic crystal, μ=85 cm^{-1} and θ_{pc}=0.92π. By integrating the power of the radiative waves over the entire device at each calculation step, we can calculate the temporal waveform of the output power of the device. The parameters used in the calculations are summarized in Table S2.

Table S2| Parameters used for 3D-CWT simulations

Symbol	Parameter	Value	Source
a	Lattice constant	274 nm	From the fabricated structure
d_{QW}	Thickness of InGaAs wells	30 nm	From the fabricated structure
n_{g}	Group refractive index	3.51	Calculated by the transfer matrix method
n_{eff}	Effective refractive index	3.43	Calculated by the transfer matrix method
Γ	Optical confinement factor	0.06	Calculated by the transfer matrix method
g_{max}	Maximum gain	3700 cm^{-1}	Calculated using k-p perturbation theory
g_0	Absorption coefficient	−4000 cm^{-1}	Calculated using k-p perturbation theory
N_{tr}	Transparency carrier density	1.3 × 10^{18} cm^{-3}	Calculated using k-p perturbation theory
Δn_{max}	Fitting parameter	-0.12	Calculated using k-p perturbation theory
N_{tr2}	Fitting parameter	4.5 × 10^{18} cm^{-3}	Calculated using k-p perturbation theory

ε	Gain suppression factor	2.45×10^{-17} cm^3	From reference [S6]
D	Diffusion constant	100 cm^2/s	Typical value; also used in our previous work [S8]
β	Spontaneous emission factor	1.0×10^{-4}	Typical value; also used in our previous work [S8]
α_{in}	Internal material loss	5.0 cm^{-1}	Typical value; also used in our previous work [S8]
τ_c	Carrier lifetime	2.0 ns	Typical value; also used in our previous work [S8]

Supplementary Note 3. Robustness of short-pulse generation in self-evolving photonic crystals

Here, we discuss the robustness of short-pulse generation in self-evolving photonic crystals. Figure S2a shows the calculated temporal waveforms of the graded photonic crystal designed in the main text (α_1=α_2=0.22 nm, β=0.11 nm) at various injection currents, where the origin of the horizontal axis (0 ns) is shifted to the moment when sufficient time (7 ns) has passed after the start of current injection. As seen in the figure, periodic pulse trains are stably obtained for a wide range of injection currents. The calculated peak power, average power, pulse width and repetition frequency of the self-pulsation as a function of injection current are shown in Fig. S2b-S2d. In Fig. S2b, the peak power is almost ten times larger than the average power owing to the equivalent *Q*-switching effect induced by the self-evolution. In Fig. S2c, the pulse width decreases as the current increases, the reason of which can be explained as follows; as the injection current (and the optical output power) increases, the speed of the stimulated-emission-

induced refractive-index change becomes faster. The resultant drastic frequency change makes the photons propagate faster along the device during the pulse generation, which leads to the shorter pulse width. The repetition frequency increases with the injection current in Fig. S2d, owing to the faster carrier accumulation in the active layer after each pulse generation.

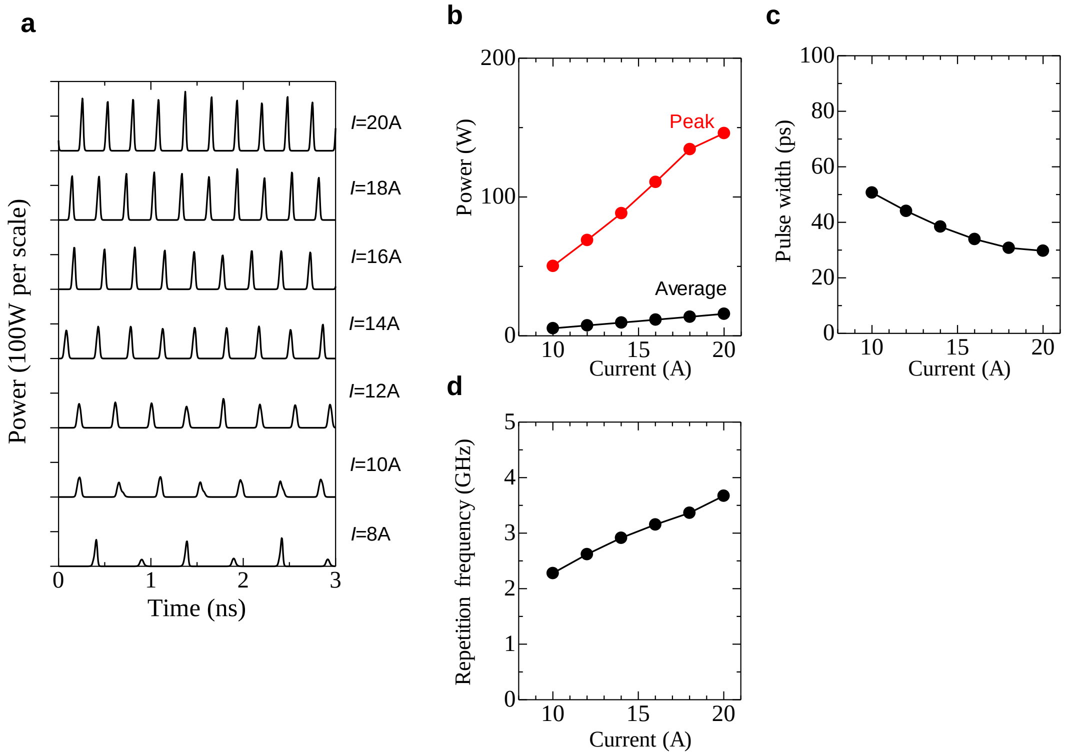

Figure S2| Calculated current dependence of pulse oscillations in self-evolving photonic crystals. a, Temporal waveforms. The origin of the horizontal axis (0 ns) is shifted to the moment when the sufficient time (7 ns) has passed after the start of current injection. **b,** Peak power and average power. **c,** Pulse width. **d,** Repetition frequency. The gradient parameters are fixed to α_1=α_2=0.22 nm and β=0.11 nm.

Next, we numerically investigate the influence of the gradient parameters (α_1, β) on the self-pulsations. As explained in the main text, α_1 represents the maximum lattice constant difference along the u-axis of the current injection region, which determines the magnitude of the mode-gap effect, while β represents the lattice constant difference inside and outside the current injection region, which determines the in-plane loss of the lasing mode outside the current injection region. Figure S3a shows the calculated transient waveforms at an injection current of 20 A for biaxially graded photonic crystals (α_1=α_2) with various designs. As shown in the figure, stable pulse trains are obtained for a wide range of gradient parameters (surrounded with a red line), which clearly shows the robustness of our design. Unstable pulsation is observed when the gradation is too small (α_1=0.055 nm), which causes the photonic mode-gap effect to be too weak to suppress multi-mode lasing. Figure S3b and S3c shows the peak power and pulse width of the stable self-pulsations shown in Fig. S3a. Higher peak powers of the self-pulsation (Fig. S3b) are obtained for larger β because the lattice constant difference inside and outside the current injection region makes the photons leak more easily into the bulk modes outside of the current injection region, which increases the cavity loss at the initial stage of the lasing and enhances the Q-switching effect. The pulse width of the self-pulsation (Fig. S3c) gradually increases as the frequency gradation α_1 increases because a larger refractive-index change is necessary to compensate the larger frequency gradation. The

minimum pulse width obtained in this simulation is 26 ps, which we consider is determined by the following three factors: (1) the differential gain of the active layer $dg(N)/dN$, which determines the speed of the stimulated emission (carrier recombination), (2) the differential refractive index change $dn(N)/dN$ of the active layer, which determines the speed of the band-edge frequency evolution, and (3) the amount of the band-edge frequency change inside the device (α_1). Although the theoretically achievable minimum pulse width is not clear at this moment, we believe that pulse widths can be further reduced by increasing $dg(N)/dN$ and $dn(N)/dN$ in the active layer.

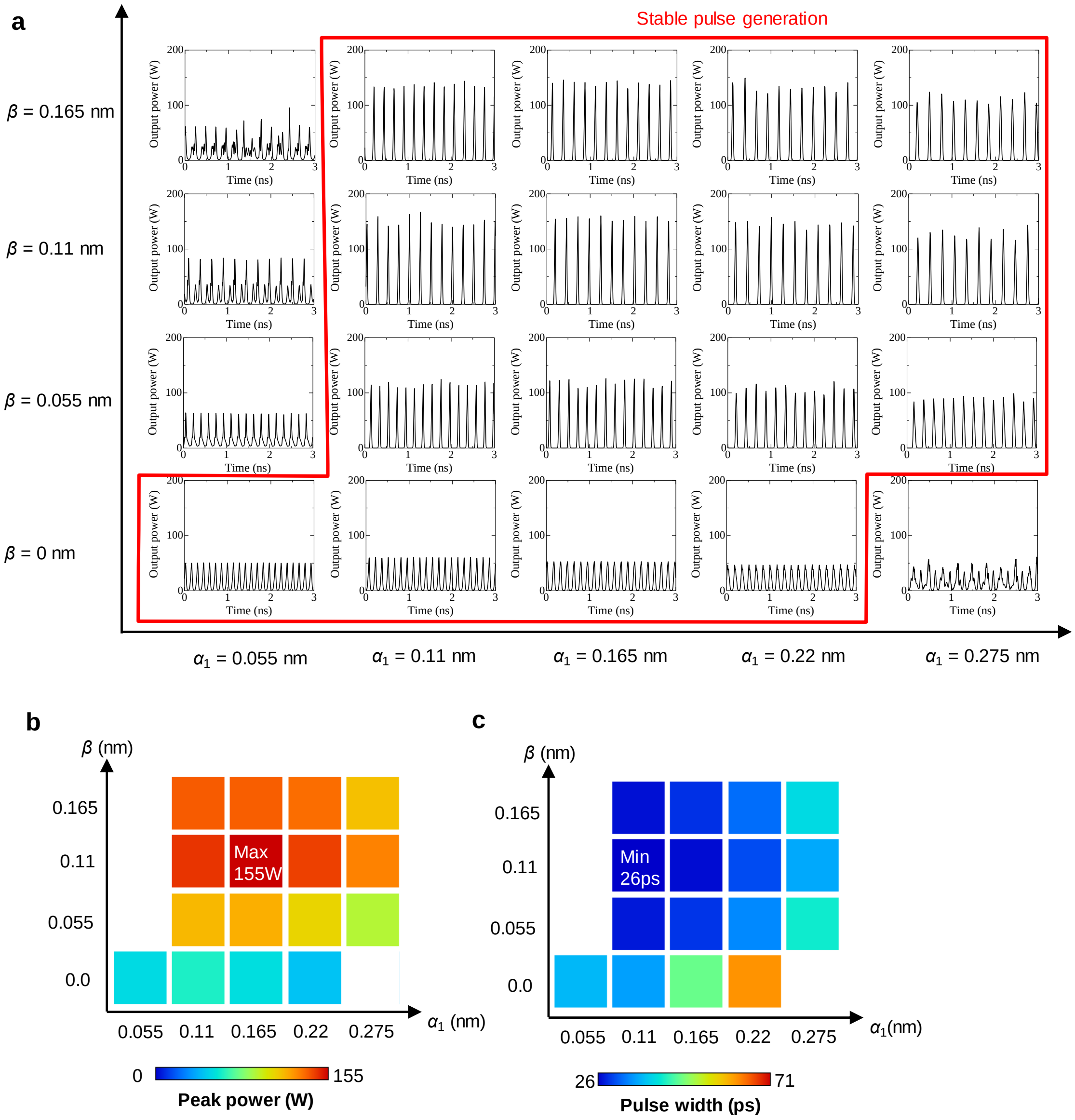

Figure S3| Transient analysis of graded photonic crystals for various gradient parameters. a, Temporal waveforms. The origin of the horizontal axis (0 ns) is shifted to the moment when sufficient time (7 ns) has passed after the start of current injection. The value of α_2 is equal to α_1 in these calculations. **b,** Peak power. **c,** Pulse width. The area encircled by the red line in panel **a** represents a parameter range in which stable pulsation is obtained.

Finally, we numerically investigate the influence of random lattice-constant fluctuations on the self-pulsations to discuss the robustness against the fabrication error. Figures S4a-S4d show the schematics of the lattice-constant distributions (α_1=α_2=0.22 nm, β=0.11 nm) with random Gaussian fluctuations with a correlation length of 20 μm and various standard deviations ($\sigma_{\text{fluc}}/\alpha_1$=0%, 3%, 6%, 9%). Figures S4e-S4h show the calculated temporal waveforms of the designed photonic crystals at 20A. The stable pulse generation is maintained even when a random Gaussian fluctuation of $\sigma_{\text{fluc}}/\alpha_1$=6% is superimposed on the designed lattice-constant distribution.

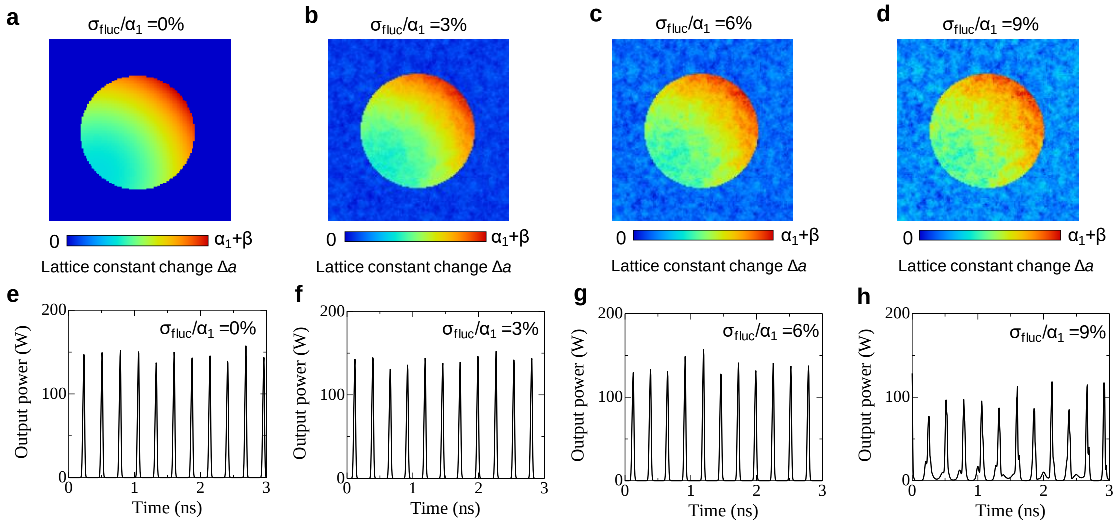

Fig. S4| Transient analysis of graded photonic crystals with random fluctuations. a-d, Schematics of lattice-constant distributions (α_1=α_2=0.22 nm, β=0.11 nm) of self-evolving photonic crystals with random Gaussian fluctuations with a correlation length of 20 μm and various standard deviations ($\sigma_{\text{fluc}}/\alpha_1$=0%, 3%, 6%, 9%). **e-h,** Calculated temporal waveforms of the designed photonic crystals with fluctuations at 20A.

Supplementary Note 4. Comparison between monoaxially and biaxially graded photonic crystals

Here, we discuss the influence of lattice constant gradation along the *v*-axis (α_2), which is perpendicular to the *u*-axis. Figure S5a shows a schematic of a monoaxially graded photonic crystal, which has a lattice constant gradation along the *u*-axis but no gradation along the *v*-axis (α_2=0). Figure S5b shows the band-edge frequency distribution along the *v*-axis after current injection and the calculated time-average carrier distribution along the *v*-axis. The carrier density at the center of the current injection section is lower than that at the edge due to spatial hole burning. As a result, the band-edge frequency distribution along the *v*-axis during the self-pulsation becomes downward convex due to the carrier-plasma effect, which makes the intensity and phase of the electric field non-uniform along the *v*-axis. Such a non-uniform distribution leads to an increase in the beam divergence angle along the *v*-axis as shown in Fig. S5c, and, in the worst case, the pulse trains themselves become unstable. On the other hand, in a biaxially graded photonic crystal, which has lattice constant gradations along both the *u*- and *v*-axes as shown in Fig. S5d (α_2=α_1), the carrier-induced refractive index change is compensated by the pre-designed parabolic frequency gradation as shown in Fig. S5e. Therefore, a uniform electric field distribution is obtained along the *v*-axis and a much narrower beam divergence angle is obtained as shown in Fig. S5f.

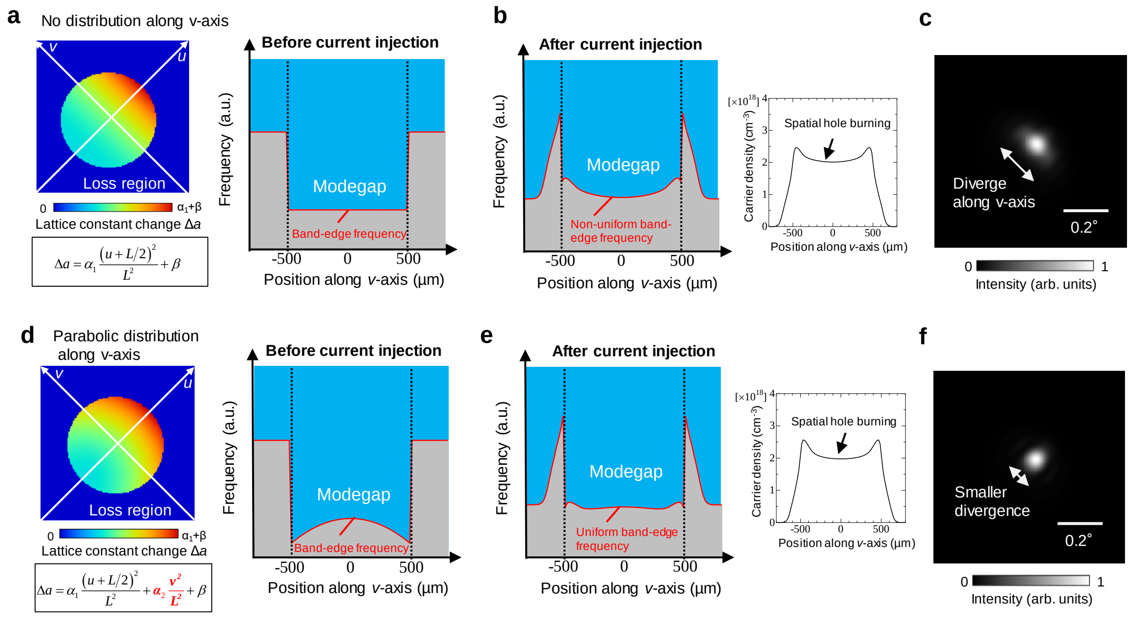

Figure S5| Numerical simulation of graded photonic crystals with monoaxial gradation (α_1=0.22 nm, α_2=0, β=0.11 nm) and biaxial gradation (α_1=α_2=0.22 nm, β=0.11 nm). a,d Schematic of two-dimensional lattice constant gradation (left panels) and band-edge frequency distributions along the *v*-axis before current injection (right panels). **b,e** Calculated band-edge frequency distributions along the *v*-axis during self-pulsation (left panels) and carrier density distributions along the *v*-axis (right panels) at an injection current of 20 A. **c,f** Calculated time-averaged far-field beam patterns at an injection current of 20 A.

We also compared the lasing characteristics of the monoaxially and biaxially graded photonic crystals in the experiment. Figures S6a and S6b show the measured streak camera images and far-field beam patterns of the fabricated monoaxially and

biaxially graded photonic crystals at an injection current of 20 A. Self-pulsation was observed in both devices. In addition, small pulses between random pairs of main pulses were observed for the monoaxial device (Fig. S6a) but not for the biaxial device, which indicates that the introduction of biaxial gradation facilitates more stable pulse oscillation. The far-field beam divergence angle of the biaxial device (Fig. S6b) was much narrower than that of the monoaxial device, which agrees well with the simulated results shown in Fig. S5. It should be noted that the measured beam pattern of the fabricated biaxial device was still elongated along its y-axis, which indicates incomplete compensation of the carrier-induced refractive-index distribution along this axis. This elongation can be reduced by optimizing the profile of the biaxial gradation.

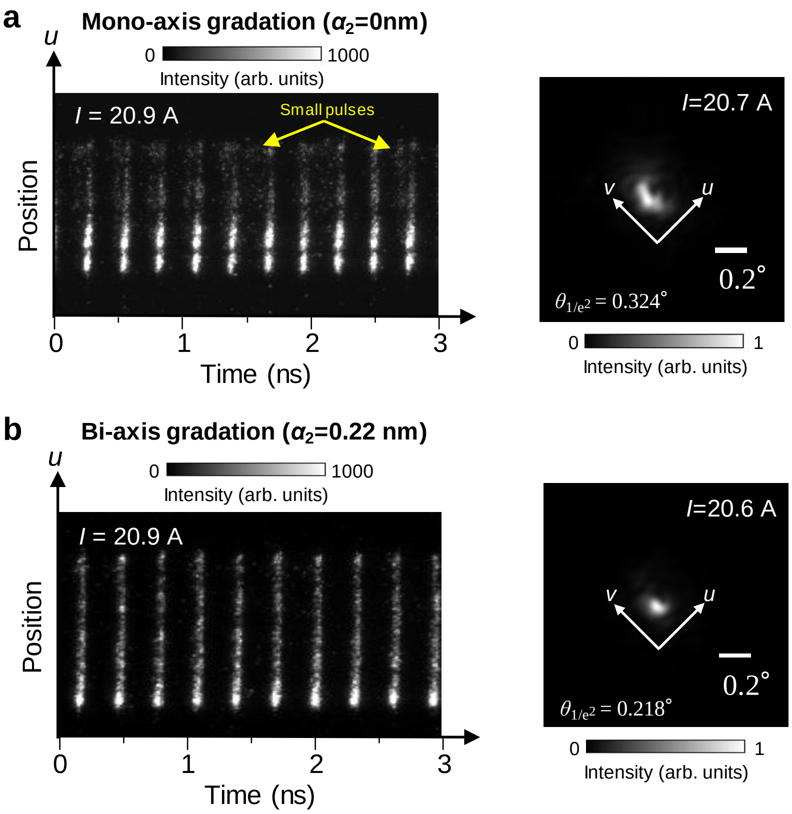

Figure S6| Characterization of self-evolving photonic crystals with monoaxial and biaxial gradations. a, Measured streak camera image and far-field beam pattern of the monoaxially graded photonic crystal (α_1=0.22 nm, α_2=0, β=0.11 nm). **b,** Measured streak camera image and far-field beam pattern of the biaxially graded photonic crystal (α_1=α_2=0.22 nm, β=0.11 nm). The divergence angles θ_{1/e^2} are average values of those evaluated at $1/e^2$ of the maximum in the x and y directions.

Supplementary Note 5. Streak camera images for various injection currents

Figure S7 shows the measured streak camera images of the fabricated biaxially graded photonic crystal (α_1=α_2=0.22 nm, β=0.11 nm) at various injection currents. Stable pulsation is obtained over a wide range of injection currents. The photons propagate in the positive direction along the *u*-axis as explained in the main text. It should be noted that when the injection current is relatively small (8.3 A), photons propagate only halfway through the device once every two pulsations. Such behaviour is also confirmed in the simulations shown in Fig. S2a, where high-peak-power pulses and low-peak-power pulses are generated in alternation at an injection current of 8 A.

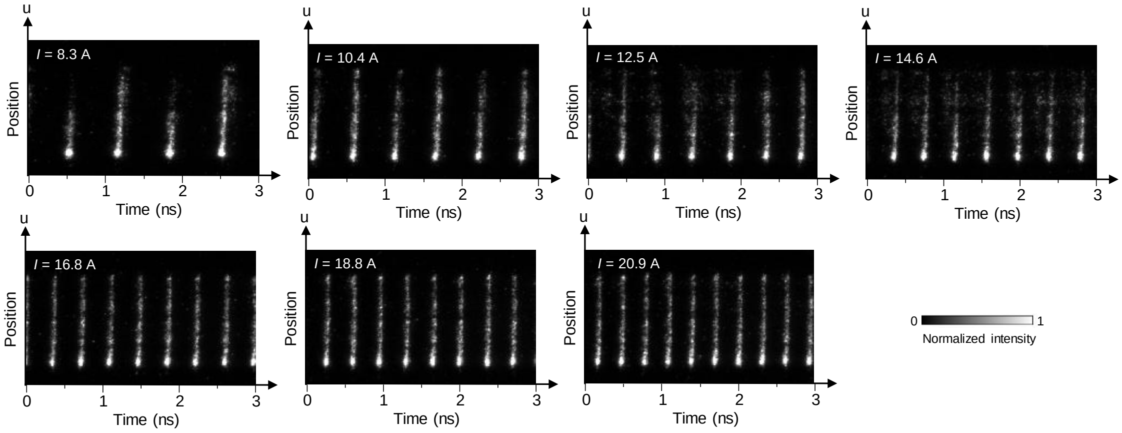

Figure S7| Measured streak camera images of the fabricated biaxially graded photonic crystal (α_1=α_2=0.22 nm, β=0.11 nm) at various injection currents.

Supplementary Note 6. External control of repetition frequency of self-pulsation

To investigate the possibility of the external triggering of our device, we

performed the transient analysis of our graded photonic crystal under the driving condition where a sinusoidal radio frequency (RF) signal is superimposed on direct current (DC). Figure S8a shows the calculated temporal waveforms of the graded photonic crystal designed in the main text (α_1=α_2=0.22 nm, β=0.11 nm) under DC of 20 A with a RF signal of 10 A_{pp} with various frequencies f_{RF}. As shown in the figure, the repetition frequency of the self-pulsation can be externally controlled by the superimposition of RF signals. Figure S8b shows the relationship between f_{RF} and the repetition frequency of the self-pulsation, where both frequencies almost coincide with each other. More precise locking of the repetition frequency may be also possible by replacing the sinusoidal RF signal with another pulsed trigger signal.

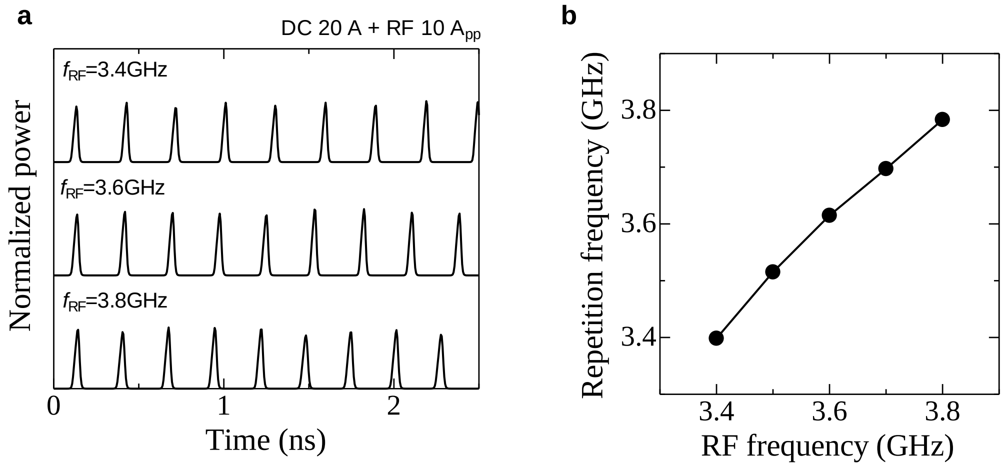

Figure S8| Calculated temporal waveforms with the superimposition of RF signals. a, Calculated temporal waveforms of self-evolving PCSELs under DC of 20A with sinusoidal RF signals with various frequencies f_{RF}. **b,** Relationship between f_{RF} and the repetition frequency of the self-pulsation.

Supplementary Note 7. Numerical simulation of pulse compression of self-evolving photonic crystals

For the simulation of instantaneous angular frequency (wavelength) of the lasing mode (Fig. 4a), we calculate the temporal derivative of the argument of the complex electric field of the radiative waves [Eq. (S9)] as follows:

$$\omega(t) = \frac{d}{dt}\left[\arg\left(E_{i,\mathrm{rad}}(t)\right)\right] \quad (i = x, y), \quad \lambda(t) = \frac{2\pi c}{\omega(t)}. \tag{S10}$$

For the simulation of the output power after second-order dispersion compensation, we first calculate the lasing spectrum by performing the Fourier transform of the complex electric field of the radiative waves [Eq. (S9)]:

$$f_i(\omega) = F\left(E_{i,\mathrm{rad}}(t)\right) \quad \left(i = x, y\right). \tag{S11}$$

By multiplying the phase compensation term of the second-order dispersion medium and taking the inverse Fourier transform, we obtain the complex electric fields of the radiative waves after dispersion compensation:

$$E_{i,\mathrm{rad,comp}}(t) = F^{-1}\left[f_i(\omega)\exp\left(i\frac{D}{2}\omega^2\right)\right] \quad \left(i = x, y\right). \tag{S12}$$

By integrating Eq. (S12) over the entire device area, we obtain the temporal waveform of the output power after dispersion compensation.

Supplementary Note 8. Design of a self-evolving photonic crystal with a larger lasing diameter

Here, we design a self-evolving photonic crystal with a larger size for higher peak-power operation. Figure S9a shows a schematic of the designed device, where the diameter of the current injection section is increased from 1.0 mm to 1.4 mm and the gradation along the *u*-axis (α_1) is increased from 0.220 nm to 0.275 nm. Figure S9b shows the calculated instantaneous wavelength change of the designed photonic crystal at an injection current of 60 A, where a peak power of 277 W is obtained before pulse compression. Figure S9c and S9d show the calculated pulse width and peak power of the self-pulsation after second-order dispersion compensation as a function of the magnitude of dispersion compensation. By adjusting the magnitude of dispersion compensation, a peak power of >1 kW with a pulse width of 6.4 ps is expected. It is also expected that the combined use of the band-edge frequency gradation proposed in this paper and two-dimensionally arranged saturable absorbers [S8] will further increase the peak power.

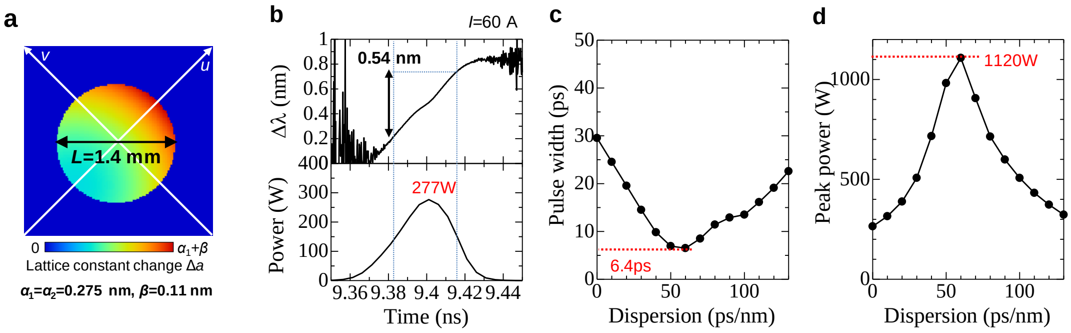

Figure S9| Design of a self-evolving photonic crystal with a larger lasing

diameter. a, Schematic of two-dimensional lattice constant distribution with a lasing diameter of L=1.4 mm. **b,** Calculated instantaneous wavelength change and output power of the designed graded photonic crystal at an injection current of 60 A. **c,d,** Calculated pulse width and peak power of the self-pulsation for the designed device as a function of the magnitude of second-order dispersion compensation.

Supplementary References

[S1] Inoue, T. *et al.* General recipe to realize photonic-crystal surface-emitting lasers with 100-W-to-1-kW single-mode operation. *Nat. Commun.* **13**, 3262 (2022).

[S2] Inoue, T. *et al.* Comprehensive analysis of photonic-crystal surface-emitting lasers via time-dependent three-dimensional coupled-wave theory. *Phys. Rev. B* **99**, 035308 (2019).

[S3] Liang, Y., Peng, C., Sakai, K., Iwahashi, S. & Noda, S. Three-dimensional coupled-wave model for square-lattice photonic crystal lasers with transverse electric polarization: a general approach. *Phys. Rev. B* **84**, 195119 (2011).

[S4] Vahara, K., Chiu, L. C., Margalit, S. & Yariv, A. On the linewidth enhancement factor α in semiconductor injection lasers. *Appl. Phys. Lett.* **42**, 631 (1983).

[S5] Chuang, S. L. *Physics of Photonics Devices 2nd Edition* (Wiley, New Jersey, 2009).

[S6] Huang, J. & Casperson, L. W. Gain and saturation in semiconductor lasers. *Opt. and*

quantum electron. **25**, 369-390 (1993).

[S7] Willatzen, W., Takahashi, T. & Arakawa, Y. Nonlinear gain effects due to carrier heating and spectral holeburning in strained-quantum well lasers. *IEEE Trans. Photon. Technol. Lett.* **4**, 682-685 (1992).

[S8] Morita, R., Inoue, T., Zoysa, M. D., Ishizaki, K. & Noda, S. Photonic-crystal lasers with two-dimensionally arranged gain and loss sections for high-peak-power short-pulse operation. *Nat. Photon.* **15**, 311–318 (2021).
