# Supplementary material for: Self-evolving photonic crystals for ultrafast photonics
Source: Nat Commun. 2023 Jan 27;14:50. doi: 10.1038/s41467-022-35599-2 (PMC9883472; doi:10.1038/s41467-022-35599-2)
Supplement: Supplementary file 3 — Lasing Reporting Summary [file 41467_2022_35599_MOESM3_ESM.pdf]

Corresponding author(s): Takuya Inoue, Susumu Noda

Lasing Reporting Summary

Nature Research wishes to improve the reproducibility of the work that we publish. This form is intended for publication with all accepted papers reporting claims of lasing and provides structure for consistency and transparency in reporting. Some list items might not apply to an individual manuscript, but all fields must be completed for clarity.

For further information on Nature Research policies, including our data availability policy, see Authors & Referees.

Experimental design

Please check: are the following details reported in the manuscript?

1. Threshold

Plots of device output power versus pump power over a wide range of values indicating a clear threshold

- [x] Yes
- [ ] No

The plot of output power versus injection current over a wide range of values can be found in Fig. 3.

2. Linewidth narrowing

Plots of spectral power density for the emission at pump powers below, around, and above the lasing threshold, indicating a clear linewidth narrowing at threshold

- [ ] Yes
- [x] No

We did not included the plots of spectral power density because the main subject of our article is the temporal response of the device. It should be noted that the measured temporal responses of short-pulse generation agree well with the simulation results, and this fact is a clear evidence of the coherent lasing oscillation.

Resolution of the spectrometer used to make spectral measurements

- [ ] Yes
- [x] No

We did not use the spectrometer for the characterization of our device.

3. Coherent emission

Measurements of the coherence and/or polarization of the emission

- [x] Yes
- [ ] No

We have measured the temporal responses and far-field patterns of our device as shown in Fig. 3, which clearly indicate the coherent lasing oscillation. We have also measured the polarization of the laser beam and confirmed that the polarization is as designed.

4. Beam spatial profile

Image and/or measurement of the spatial shape and profile of the emission, showing a well-defined beam above threshold

- [x] Yes
- [ ] No

We have measured the far-field patterns of our device at an injection current of 20 A (~5.0 Ith) and included the result as Fig. 3g.

5. Operating conditions

Description of the laser and pumping conditions
Continuous-wave, pulsed, temperature of operation

- [x] Yes
- [ ] No

We have described these information in Method section.

Threshold values provided as density values (e.g. W cm^{-2} or J cm^{-2}) taking into account the area of the device

- [x] Yes
- [ ] No

Threshold current of our device is ~0.63kA/cm^2 , which can be calculated from the threshold current (~5A, Fig. 3d) and the device diameter (1mm).

6. Alternative explanations

Reasoning as to why alternative explanations have been ruled out as responsible for the emission characteristics
e.g. amplified spontaneous, directional scattering; modification of fluorescence spectrum by the cavity

- [x] Yes
- [ ] No

Measured short-pulse high-peak-power temporal waveforms agree well with the corresponding simulation results, which clearly validate the short-pulse lasing oscillation of our device.

7. Theoretical analysis

Theoretical analysis that ensures that the experimental values measured are realistic and reasonable
e.g. laser threshold, linewidth, cavity gain-loss, efficiency

- [x] Yes
- [ ] No

Detailed simulation results are provided in Fig. 2 and Supplementary Section 3.

8. Statistics

Number of devices fabricated and tested

- [x] Yes
- [ ] No

We have measured two devices with different gradient parameters, and we have included the measured results of both devices in Supplementary Section 4.

Statistical analysis of the device performance and lifetime (time to failure)

- [x] Yes
- [ ] No

No aging test was performed, but no failure has been observed, at least in repeated measurements with varying injection currents as shown in Supplementary Section 5.
